# Repair of an iatrogenic lacrimal fistula: a case report

**DOI:** 10.11604/pamj.2024.48.105.42226

**Published:** 2024-07-15

**Authors:** Oumayma Elmansouri, Zineb Algouti, Houda Bezza, Elhoussine Ait Lhaj, Mohamed Kriet, Fouad Elasri

**Affiliations:** 1 Department of Ophthalmology, Avicenna Military Hospital, Marrakesh, Morocco

**Keywords:** Lacrimal fistula, epiphora, stripping, case report

## Abstract

Lacrimal cutaneous fistula is an uncommon yet clinically significant condition leading to persistent epiphora, recurrent infections, and significant patient discomfort. It can be congenital or acquired. Timely identification and appropriate surgical management are crucial to alleviate symptoms and restore normal lacrimal system functionality. This case report describes the presentation and surgical management of an iatrogenic lacrimal cutaneous fistula in a 68-year-old patient. The surgical intervention involved a novel stripping technique, which successfully resolved the fistula without requiring special instruments. The patient’s post-operative recovery was positive, with complete closure of the fistulous tract and restoration of normal lacrimal system function. This report underscores the efficacy of the stripping technique for managing iatrogenic lacrimal fistulas and suggests its consideration for similar cases.

## Introduction

Lacrimal cutaneous fistula is an uncommon yet clinically significant condition characterized by an abnormal passage between the inferior lacrimal canaliculus and the skin. It can arise due to various factors, including congenital anomalies, trauma, inflammatory processes, or iatrogenic causes [1,2]. The presence of a lacrimal cutaneous fistula can lead to persistent epiphora, recurrent infections, and significant patient discomfort. Timely identification and appropriate surgical management are crucial to alleviate symptoms and restore normal lacrimal system functionality. The management of lacrimal cutaneous fistulas poses a challenge to ophthalmologists and oculoplastic surgeons due to the complex anatomy of the lacrimal system and the diverse presentations of such fistulas. While conservative management and observation may be considered for certain cases, surgical intervention is often necessary to achieve a definitive and long-lasting solution [3]. We present a case of iatrogenic lacrimal cutaneous fistula in a 68-year-old patient and describe the surgical management approach employed to address this challenging condition.

## Patient and observation

**Patient information:** we report a case of lacrimal cutaneous fistula in a 68-year-old patient without any significant medical history.

**Figure 1:**
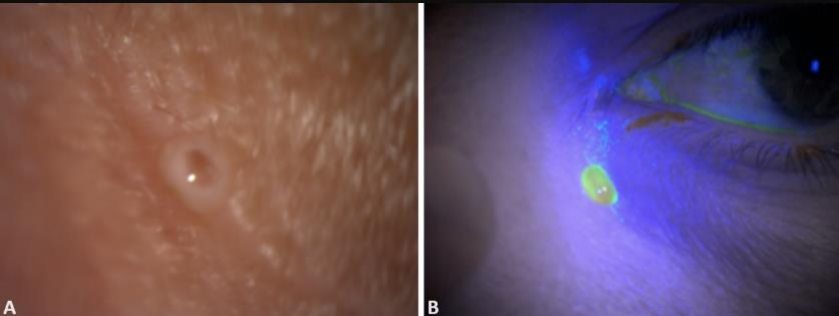
A) diagnosis of the lacrimal fistula on the left; B) clear lacrimation from the cutaneous fistula, on the right; confirmation with fluorescein test

**Clinical findings:** on examination, a skin fistula was observed at the lower eyelid level, accompanied by clear lacrimation. Additionally, a positive fluorescein test confirmed the presence of communication between the lower lacrimal canaliculus and the skin (Figure 1).

**Diagnostic assessment:** during the examination of the tear ducts, probing was conducted to assess the integrity of the lacrimal system. Probing of the inferior tear duct revealed no bone contact, indicating an obstruction along the path of the lacrimal passage. Subsequently, a lavage procedure was performed by irrigating the tear duct with saline solution. The results demonstrated the presence of saline reflux through the cutaneous fistula with no passage of the saline solution into the throat, indicating a direct communication between the inferior lacrimal canaliculus and the external skin. Notably, the probing and lavage of the superior tear duct were normal.

**Timeline of current episode:** the fistula appeared one month after the first dacryorhinocystostomy and was examined six months later for surgical correction.

**Diagnosis:** iatrogenic lachrymal fistula.

**Figure 2:**
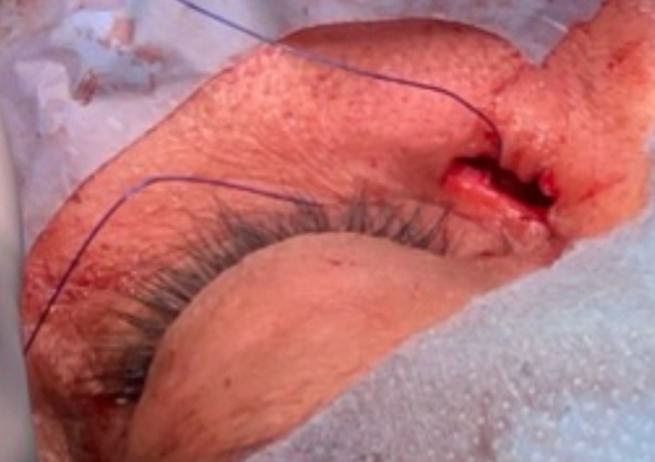
a nylon suture thread passed through the cutaneous opening of the fistula until it emerged from incision following the path of the fistula

**Therapeutic intervention:** we decided to manage the case by doing a stripping of the fistula and revising the dacryorhinocystostomy. The patient was prepared under general anesthesia, with meticulous attention to sterile protocols during the prepping and draping of the patient's face. Employing a 15-blade scalpel, an incision was made in the skin, and the fibers of the orbicularis oculi muscle were meticulously separated until reaching the periosteum of the anterior lacrimal crest. A 1-0 nylon suture thread was passed through the cutaneous opening of the fistula until it emerged from the incision following the path of the fistula (Figure 2). The thread was then securely knotted. Subsequently, the surgeon, situated at the cutaneous opening side, exerted traction on the thread, effectively extracting the fistula. Continuing the procedure, the periosteum was extensively elevated anteriorly off the nasal bone using Freer elevators. Kerrison rongeurs were employed to enlarge the osteotomy from the first surgery. A bicanalicular silicone tube was introduced through the superior and inferior punctums into the nasal fossae, and subsequently sutured to the nasal mucosa. The orbicular muscle was sutured with 8-0 vicryle, followed by the skin being meticulously closed with 6-0 prolene.

**Follow-up and outcomes:** following surgery, the patient was discharged, instructed to rest for one week and prescribed oral antibiotics. Skin sutures were removed one week postoperatively and the silicone tube was removed 8 weeks after surgery.

**Patient perspective:** at the post-operative check-up, the patient was satisfied. The lacrimal passages were permeable and the skin opening had healed well.

**Informed consent:** consent was obtained, and the patient was made aware that her medical records would be kept confidential.

## Discussion

Lacrimal fistulae may manifest as congenital or acquired anomalies. Symptomatic congenital fistulae are commonly linked to underlying transient functional or anatomical nasolacrimal duct obstructions. Management approaches vary based on symptomatology, with observation sufficing for asymptomatic cases, while symptomatic instances or those causing cosmetic concerns may necessitate fistulectomy to address underlying pathology [2,3]. Acquired lacrimal fistulae (ALF), on the other hand, commonly result from the spontaneous rupture of untreated lacrimal abscesses, traumatic incidents, or iatrogenic factors such as poorly performed incisions and drainage procedures. These acquired fistulae can manifest anywhere along the lacrimal drainage pathway, exhibiting irregularities and often attaining larger sizes accompanied by peri-fistulous soft tissue changes [1,4].

Our patient presented with acquired lacrimal fistula secondary to probable poorly-performed probing of the inferior canaliculus. The literature documents various techniques for managing acquired lacrimal fistulae, including strategies such as allowing healing through secondary intention, incising along the fistulous pathway with subsequent tract excision, and performing fistulectomy in conjunction with a skin incision and dacryocystorhinostomy [5]. Most of the acquired fistulae heal by themselves irrespective of the nature, size, and location and need no additional treatment in the form of fistulectomy as they do not appear to be lined with squamous epithelium. This makes the closure easier and quicker than congenital lacrimal fistulae with a skin lined tract [4]. Ali *et al*. [6] conducted a comparative analysis of surgical outcomes between congenital and acquired lacrimal fistula cases. In both groups, treatment involved fistulectomy and bicanalicular silicone tube intubation for nasolacrimal duct obstruction (NLDO), resulting in complete closure of the fistulous tract in all cases. To our knowledge, the stripping technique used in our case was not described in other cases of lacrimal fistula but the result in our patient is promising. It is an easy and quick technique that don´t require special instruments that should be considered in other cases of lacrimal fistula.

## Conclusion

This case report contributes valuable insights into the efficacy and outcomes of fistula stripping as a therapeutic intervention for iatrogenic lacrimal fistula. Our findings underscore the success of the procedure in achieving complete closure of the fistulous tract, thus addressing the sequelae of iatrogenic complications. While our study sheds light on the immediate outcomes, longitudinal follow-up studies may provide further insights into the long-term efficacy and potential recurrence rates associated with the shipping in iatrogenic lacrimal fistula cases.
